# Mr. Purton, on the Contagion of the Influenza

**Published:** 1804-01-01

**Authors:** T. Purton

**Affiliations:** Alcester


### *To the Editors of the Medical and Physical Journal.*

Gentlemen,

On reading over the arguments and opinions respecting the late Epidemic, published in your Journal, I cannot fail being surprised to find such opposite sentiments; one party believing it to be derived from the air; the other, that it is contagious, or communicable from person to person. Soon after the commencement of this disease I formed my own opinion from what I thought accurate observation, and I have had no reason to alter it. It appeared in this town and neighbourhood the beginning of March, in several families nearly at one and the same time, and disappeared as suddenly, the latter end of May. I took notice that, at first, one or two in a numerous family would have it, and the rest escape; but after the disease became more general, it put on a more contagious aspect, which I conceived was caused by a greater number of Constitutions being attacked, generating thereby *another morbid influence, very different from the first.* For, I believe it cannot well be denied, that contagious effluvia may thus be generated, and that also some particular constitutions are more prone to run into those states productive of it, than others. Upon this I grounded my opinion, and I am clearly convinced that the advocates for original Contagion cannot establish their point; for where it was *apparently* contagious, it was so by accident; the poison already generated in some constitutions had diffused itself, breaking down that healthy barrier which hitherto had resisted the atmospheric influence, and thereby received the epidemic under a new form; and, in this manner, we can easily account for the diversity of opinions that have been advanced in this interesting controversy. In regard to the treatment, some have found the use of the lancet altogether inadmissible, whilst others have repeated the operation with evident good effects. How can we reconcile this contrariety in practice, if we are to suppose that in both instances the patients were labouring under the sedative effects of human contagion?

I do not in the least flatter myself, that any thing which I have now advanced will meet the ideas of those gentlemen who have published such direct and opposite sentiments; but as I am clearly of opinion that the late epidemic, termed Influenza, was no more contagious *in itself* than a common catarrh, I consider it as a bounden duty I owe to you, Gentlemen, as a Reader of your Journal, and to the Public, to make those opinions known, which have produced such complete conviction in my own mind.

I am, &c.

*Alcester, Nov.* 12, 1803. T. PURTON.

P. S. As the cause which produced these changes in the atmosphere, appear to have operated very generally over this side of the globe, having its origin many degrees eastward of us, we cannot reasonably suppose that human effluvia floated in the air, and was *rapidly* wafted to us from countries so remote; it is the rapidity with which it passed from one side of the island to the other, which completely does away the argument in favour of contagion; and, indeed, as we are led to believe, from great authority, that when contagious matter is conveyed by the air, it has *never been found to act far from the sources from whence it arose,* (Cullen, First Lines, vol. 1, p. 82) how can we suppose for a moment that the late epidemic had its origin from human contagion, as we well know that it appeared in France and other countries before it made us a visit?

Account

